# Editorial: SARS-CoV-2 in neurodegenerative diseases

**DOI:** 10.3389/fnins.2024.1360234

**Published:** 2024-01-16

**Authors:** Gonzalo Emiliano Aranda-Abreu, Alfonso Carreón-Rodriguez, Sonia Zuñiga, David Pozo

**Affiliations:** ^1^Instituto de Investigaciones Cerebrales, Universidad Veracruzana, Xalapa, Mexico; ^2^Instituto Nacional de Salud Pública, Cuernavaca, Morelos, Mexico; ^3^National Center for Biotechnology, Spanish National Research Council (CSIC), Madrid, Spain; ^4^Department of Medical Biochemistry, Molecular Biology and Immunology, Faculty of Medicine, University of Seville, Seville, Spain

**Keywords:** SARS-CoV-2, neurodegeneration, brain, disease, neuroinflammation

In the month of March in the year 2020, an alarming pandemic triggered by the SARS-CoV-2 virus, which leads to the development of the notorious COVID-19 disease, was officially declared. The consequences of this coronavirus have been utterly devastating, resulting in the unfortunate demise of countless individuals due to the severe deterioration experienced both in the pulmonary as well as systemic aspects of their health. Regrettably, it has been observed that the elderly population and those individuals with underlying health conditions, commonly referred to as comorbidities, have proven to be the most susceptible and vulnerable to the detrimental effects of this virus. Astonishingly, statistics have estimated that an astonishing number exceeding 70 million people across the globe have been diagnosed with the SARS-CoV-2 virus, including individuals diagnosed with neurodegenerative diseases such as Alzheimer's, who happen to be at the highest risk for both hospitalization and mortality.

The mechanisms and pathways through which the virus infiltrates and impacts the nervous system have been subjects of extensive research and scientific inquiry. One of the primary proposed pathways suggests that the virus is capable of directly infecting neurons, thereby triggering a cascading sequence of events that ultimately leads to the induction of various inflammatory agents. Interestingly, these inflammatory agents are generated as a result of the systemic inflammation that transmits to the brain. Consequently, it becomes increasingly evident that individuals diagnosed with neurodegenerative diseases must be given heightened attention and care, as there is a growing body of evidence suggesting that the presence of the coronavirus can significantly exacerbate the progression and severity of such diseases. Consequently, it becomes absolutely imperative to actively seek and develop effective therapeutic interventions and treatment modalities specifically tailored for individuals diagnosed with neurodegenerative diseases, with the ultimate goal of mitigating and decelerating the adverse effects caused by the SARS-CoV-2 virus.

The COVID-19 pandemic has presented an unparalleled array of challenges, the implications of which on the central nervous system have emerged as a critical area of investigation. Within this editorial, we will delve into a plethora of studies that shed light on the intricate and interconnected nature of the relationship between the SARS-CoV-2 virus and the alterations it induces within the cerebral realm.

The following articles are part of this topic.

1. *Brain Cortical Alterations in COVID-19 Patients with Neurological Symptoms* (Sanabria-Diaz et al.).
2. Studies examining cortical alterations in COVID-19 patients with neurological symptoms reveal the diverse range of manifestations the virus can induce within the brain. From changes in connectivity to cognitive impairments, understanding these alterations is crucial for guiding clinical interventions.
3. *Risk and Prognostic Factors for SARS-CoV-2 Infection in a Spanish Multiple Sclerosis Population during the First 5 Waves* (Pilo De La Fuente et al.).
4. The intersection of SARS-CoV-2 and multiple sclerosis in the Spanish population highlights the need to identify risk and prognostic factors. This knowledge is essential for tailoring prevention and treatment strategies in this specific cohort.
5. *SARS-CoV-2, Long COVID, Prion Disease, and Neurodegeneration* (Zhao et al.).
6. The intricate relationship between SARS-CoV-2, long COVID, prion diseases, and neurodegeneration poses urgent questions. How does the virus influence prion pathways, and what are the implications for long-term neurodegeneration?
7. *Late Neurological Consequences of SARS-CoV-2 Infection: New Challenges for the Neurologist* (Korchut and Rejdak).
8. Understanding the late neurological consequences of SARS-CoV-2 infection presents novel challenges for neurologists. From persistent symptoms to cognitive issues, specialized attention and adaptive management strategies are required.
9. *SARS-CoV-2-Specific Antibody Responses Following BNT162b2 Vaccination in Individuals with Multiple Sclerosis Receiving Different Disease-Modifying Treatments* (Lambrianides et al.).
10. The variability in SARS-CoV-2-specific antibody responses post-BNT162b2 vaccination in multiple sclerosis patients prompts questions about the efficacy of diverse disease-modifying treatments.
11. *Adamantanes for the Treatment of Neurodegenerative Diseases in the Presence of SARS-CoV-2* (Butterworth).
12. Exploring adamantanes as a potential treatment for neurodegenerative diseases in the context of SARS-CoV-2 underscores the need for innovative and multifaceted therapeutic strategies.
13. *The Link between SARS-CoV-2-Related Microglial Reactivity and Astrocyte Pathology in the Inferior Olivary Nucleus* (Madden et al.).
14. Investigating the connection between SARS-CoV-2-related microglial reactivity and astrocyte pathology in specific brain regions provides unique insights into the mechanisms behind neurological manifestations of the virus.
15. *COVID-19: A Modern Trigger for Guillain-Barré Syndrome, Myasthenia Gravis, and Small Fiber Neuropathy* (Gomez et al.).
16. The association between COVID-19 and neuromuscular syndromes raises questions about pathogenesis and necessitates heightened vigilance in affected patients.
17. *Long-Lasting Neutralizing Antibodies and T Cell Response After the Third Dose of mRNA Anti-SARS-CoV-2 Vaccine in Multiple Sclerosis* (Maglione et al.).
18. The persistence of neutralizing antibodies and T cell responses after the third dose of mRNA anti-SARS-CoV-2 vaccine underscores the importance of tailored vaccination strategies for individuals with multiple sclerosis.
19. *The Determinants of COVID-Induced Brain Dysfunctions After SARS-CoV-2 Infection in Hospitalized Patients* (Yasir et al.).
20. Identifying the determinants of COVID-induced brain dysfunctions after SARS-CoV-2 infection in hospitalized patients is crucial for enhancing care and rehabilitation.

In conclusion, the COVID-19 pandemic has brought about a myriad of challenges, particularly in relation to the central nervous system. The intricate connections between the SARS-CoV-2 virus and cerebral alterations necessitate comprehensive investigations in order to enhance our understanding of the underlying mechanisms and develop effective interventions. By addressing the various aspects discussed in this editorial, we can work toward mitigating the impact of COVID-19 on the central nervous system and improving patient outcomes.

## Author contributions

GA-A: Writing – original draft, Writing – review & editing. AC-R: Writing – original draft, Writing – review & editing. SZ: Writing – original draft, Writing – review & editing. DP: Writing – original draft, Writing – review & editing.

